# Association Among Serum Vitamin D Levels, Visual Field Alterations, and Optical Coherence Tomography Parameters: A Clinical Correlation Study

**DOI:** 10.3390/life16010085

**Published:** 2026-01-06

**Authors:** Tudor-Corneliu Tarași, Mihaela-Madalina Timofte-Zorila, Filippo Lixi, Mario Troisi, Giuseppe Giannaccare, Luminița Apostu, Ecaterina Anisie, Livio Vitiello, Daniel-Constantin Brănișteanu

**Affiliations:** 1 Department of Ophthalmology, Grigore T. Popa University of Medicine and Pharmacy, 700115 Iasi, Romania; 2 Department of Ophthalmology, Cai Ferate Clinical Hospital, 700506 Iasi, Romania; 3 Eye Clinic, Department of Surgical Sciences, University of Cagliari, Via Università 40, 09124 Cagliari, Italy; 4 Ophthalmology Unit, Salerno University Hospital, Via San Leonardo, 84131 Salerno, Italy; 5 Saint Spiridon Emergency Hospital, 700111 Iasi, Romania; 6 Eye Unit, Luigi Curto Hospital, Azienda Sanitaria Locale Salerno, 84035 Polla, Italy

**Keywords:** vitamin D deficiency, retinal microvasculature, optical coherence tomography angiography (OCTA), ganglion cell complex (GCC), visual field sensitivity, retinal perfusion, neuroretinal integrity

## Abstract

Vitamin D deficiency is increasingly recognized as a systemic factor influencing retinal health through inflammatory, neuroprotective, and vasculotropic pathways. Evidence regarding early retinal alterations in otherwise healthy adults remains limited. This cross-sectional study evaluated 120 eyes from 60 healthy adults stratified by serum 25(OH)D levels into <30 ng/mL (n = 60) and ≥30 ng/mL (n = 60). All subjects underwent optical coherence tomography (OCT), OCT angiography (OCTA), visual field testing, and contrast sensitivity assessment. Central macular thickness (CMT), ganglion cell complex (GCC) thickness, and perfusion density in the superficial and deep capillary plexuses (SCP, DCP) were compared between groups. Vitamin-D-insufficient eyes showed significantly reduced CMT (267.66 ± 13.31 µm vs. 274.69 ± 14.96 µm; *p* = 0.035). GCC thinning was significant only in the inner inferior nasal sector (70.7 ± 13.14 µm vs. 76.45 ± 12.12 µm; *p* = 0.030), whereas other GCC sectors were comparable between groups. Perfusion density was lower in the DCP across whole, inner, and outer regions (all *p* < 0.001) and in the SCP inner (*p* = 0.027) and outer (*p* = 0.009) regions, while whole SCP did not differ (*p* = 0.065). FAZ area was numerically larger in vitamin-D-insufficient eyes but was not statistically different (*p* = 0.168). Functionally, retinal sensitivity decline was greater in vitamin-D-insufficient eyes (−2.89 ± 1.29 dB vs. −2.16 ± 1.04 dB; *p* = 0.003), and mean central sensitivity was lower (*p* = 0.010), whereas contrast sensitivity did not differ between groups. Serum vitamin D levels < 30 ng/mL are associated with early, subclinical, structural and microvascular retinal alterations in healthy adults, supporting a potential role of hypovitaminosis D as a modifier of retinal integrity.

## 1. Introduction

Vitamin D is a fat-soluble pre-hormone synthesized from cholesterol and plays a central role in calcium–phosphorus homeostasis, cellular metabolism, and immunomodulation. In recent years, vitamin D deficiency has emerged as a major global public health concern, with particularly high prevalence reported across the European population [1,2]. Serum concentrations below 30 ng/mL are generally considered insufficient and have been associated with increased risk of several systemic disorders [3].

Beyond its classical endocrine functions, accumulating evidence suggests that vitamin D also contributes to ocular health. Low vitamin D levels have been linked to diabetic retinopathy (DR), age-related macular degeneration (AMD), and glaucoma [4,5]. Experimental studies indicate that vitamin D may exert protective ocular effects by reducing oxidative stress and inflammation in retinal pigment epithelial (RPE) cells, modulating endothelial function, and exerting anti-angiogenic activity [6,7,8].

Advances in retinal imaging have enabled detailed evaluation of both retinal structure and microvascular networks. Optical coherence tomography (OCT) allows high-resolution assessment of retinal layers, while OCT angiography (OCTA) provides non-invasive visualization of retinal and choroidal microvasculature [9]. Complementary to structural imaging, visual field (VF) testing remains an essential tool for assessing neuro-retinal functional integrity [10].

Although several studies have examined the association between vitamin D status and macular structural changes [11,12], evidence regarding the impact of vitamin D levels on retinal function—particularly in healthy individuals—remains limited. Moreover, few studies have simultaneously evaluated structural and microvascular parameters alongside functional outcomes.

The aim of this study was therefore to investigate whether serum vitamin D levels influence retinal structure, microvascular perfusion, and visual function in healthy subjects, using a multimodal assessment combining OCT, OCTA, and VF testing.

## 2. Materials and Methods

In this cross-sectional, controlled study patients were recruited at Ochidoc Clinic of Iasi, Romania, between June and November 2025. The study adhered to the tenets of the Declaration of Helsinki and was approved by the Ethics Committee of University of Medicine and Pharmacy Gr. T. Popa, Iasi (Nr. 609/10 June 2025). Written informed consent was obtained from all participants.

### 2.1. Study Population

The study included participants aged 18–50 years with insufficient serum vitamin D levels and no systemic or ocular diseases. A control group was composed of healthy individuals with sufficient serum vitamin D levels who presented to the ophthalmology clinic for routine eye examinations. Serum vitamin D concentrations were measured using an electrochemiluminescence immunoassay on the Roche Cobas e601 analyzer (Roche Diagnostics, Mannheim, Germany) with Vitamin D Total III kits (Roche Diagnostics, Germany). Peripheral venous blood samples were collected in the morning. Vitamin D status was categorized as sufficient (>30 ng/mL) or insufficient (<30 ng/mL).

Only subjects with best-corrected visual acuity (BCVA) of 0.0 LogMAR were included. Individuals with systemic diseases such as diabetes mellitus, arterial hypertension, thyroid dysfunction (hypothyroidism or hyperthyroidism), dyslipidaemia, vasculitis, rheumatologic disorders, or neurological disease were excluded. Participants with any ocular pathology—including retinal vascular disease, maculopathies, glaucoma, optic nerve disorders, uveitis, cataract, amblyopia, nystagmus, or a history of ocular surgery—were also excluded. Additional exclusion criteria included a spherical equivalent beyond ±3.00 D, astigmatism > 1.50 D, or an axial length outside 21.50–24.50 mm. Individuals with a body mass index (BMI) < 18 kg/m^2^ or >25 kg/m^2^, as well as those using vitamin D analogs, lutein-based supplements, or medications known to affect vitamin D metabolism, were likewise excluded.

### 2.2. Data Acquisition

After the eligibility screening, participants fulfilling study criteria were analyzed for the collection of the following data: age, sex, body mass index (BMI), BCVA, refraction measurement, slit lamp biomicroscopy, indirect ophthalmoscopy, intraocular pressure (IOP) measurement with Goldmann applanation tonometry, contrast sensitivity testing with Pelli-Robson charts at 100 cm distance, OCT and OCTA exams (RS-3000 Advance AngioScan, Nidek Co., Ltd., Gamagori, Japan) and VF test (Optopol PTS 920 perimeter, Optopol Technology, Zawiercie, Poland).

**Figure 1:**
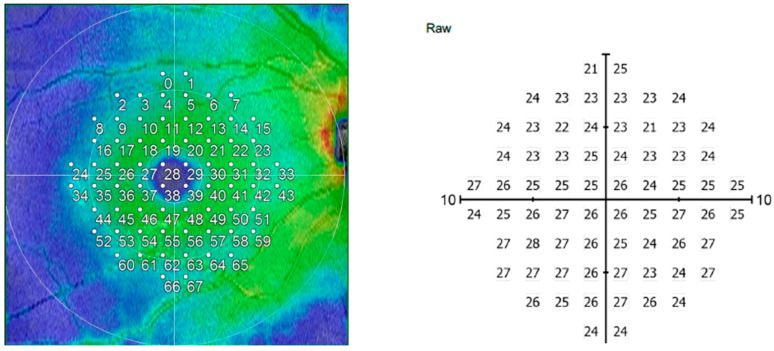
Representation of data points of the right eye of an OCT macula map scan and raw data of central macular visual field with equivalent disposition of points.

All examinations were performed between 9:00 am and 12:00 pm. Scans with poor signal strength or significant segmentation errors were excluded. Macular map scans (9 × 9 mm) were obtained to quantify retinal nerve fiber layer (RNFL) and ganglion cell complex (GCC) thicknesses. Retinal measurements were extracted using the ETDRS grid, which divides the macula into central (1 mm), inner (1–3 mm), and outer (3–6 mm) rings, with the inner and outer rings further subdivided into superior, inferior, nasal, and temporal quadrants. Mean values for each sector were calculated for statistical analysis. Central macular thickness (CMT) was defined as the mean retinal thickness within the central 1 mm ETDRS ring, corresponding to the value provided by the most central circle of the ETDRS thickness grid. In contrast, the mean central points parameter represented the average thickness of the individual central data points sampled by the 68-point M-10 grid of the NIDEK RS-3000, which is designed to closely mirror the topographic arrangement of macular visual field test locations around the foveal center. The mean paracentral points reflected the average thickness of the points immediately surrounding the central region within the same M-10 grid, providing a quantitative measure of the paracentral macular area (Figure 1).

Optic nerve evaluation was performed using disk map scans (6 × 6 mm), and RNFL thicknesses were measured globally, by quadrants, and by clock hours.

**Figure 2:**
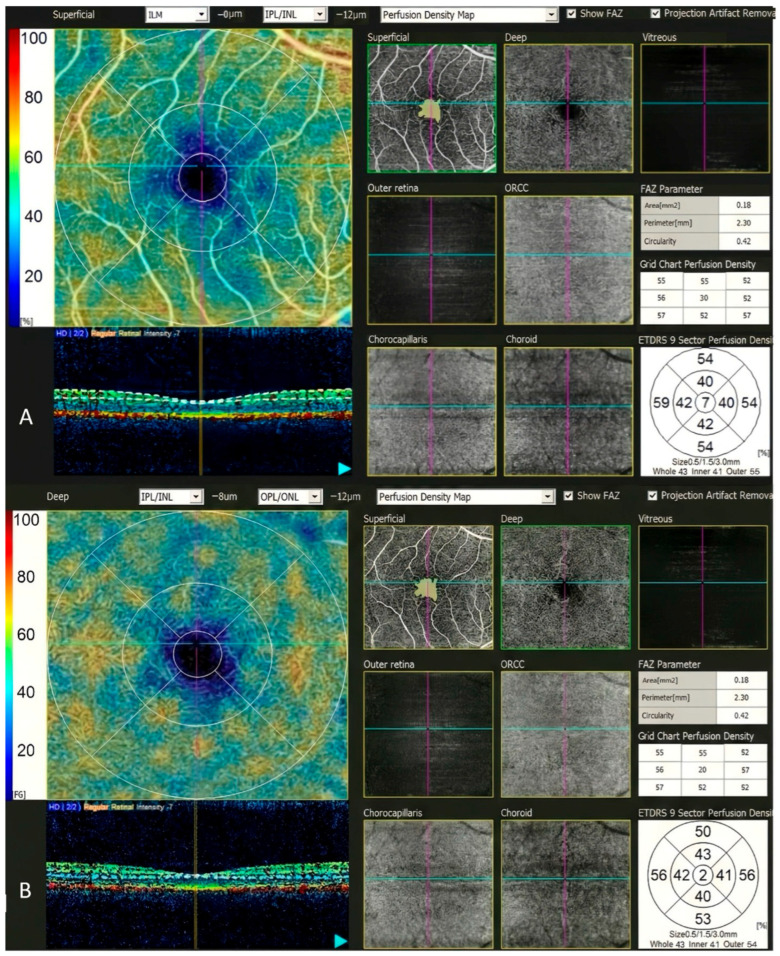
OCTA images of SCP (**A**) and DCP (**B**), and relevant metrics of a representative patient with vitamin D level of 32.14 ng/mL and with optimal scan quality [signal quality index (SQI) 5/5 and signal strength index (SSI) 10/10], shown for segmentation topology and slab boundaries.

OCTA scans (3 × 3 mm) were acquired, and retinal layer thicknesses were automatically calculated by the device. To ensure measurement reliability and reduce random measurement error, all OCTA parameters were obtained as the average of 3 repeated 3 × 3 mm scans per eye, acquired in the same time interval and with consistent segmentation and signal strength, according to quality-control criteria. The superficial capillary plexus (SCP) was defined as the slab extending from the internal limiting membrane to 12 µm below the inner plexiform layer, while the deep capillary plexus (DCP) was defined as the layer extending from 8 µm below the inner nuclear layer to 12 µm below the outer nuclear layer. En face images of these slabs were analyzed, and data on microvascular architecture, vessel density indices, and foveal avascular zone (FAZ) characteristics were recorded. Perfusion density was automatically calculated by the device as the percentage of perfused vessel pixels within the designated region, using a binarized flow map and a proprietary algorithm (Figure 2).

VF test was performed with the macular testing protocol, which evaluates 68 test points within the central visual field.

The analysis included global indices such as mean deviation (MD), hemifield mean deviation (hMD), hill of vision at 10 degrees (HOV@10°), and foveal threshold. Test points were analyzed individually or grouped topographically by quadrants, and as central or paracentral zones, to assess localized functional variations. Raw sensitivity values and mean sensitivity for each quadrant were recorded. The four central foveal points and the foveal threshold were classified as central, while adjacent points were designated as paracentral; mean values were calculated for each zone. Unreliable visual fields were excluded.

### 2.3. Statistical Analysis

Statistical analysis was conducted using SPSS for Macintosh software (version 30.0.0.0, SPSS, Inc., USA). Numerical data are shown as mean ± standard deviations (SDs). Normality of the data distribution was assessed using the Shapiro–Wilk. Differences between the two groups were tested with an independent-samples *t*-test. Chi-squared test was employed to compare categorical variables. The relationship between vitamin D serum levels and other numerical data was evaluated using Pearson correlation test. Correlation coefficients are reported together with their 95% confidence intervals to convey the precision of the estimates. To account for multiple testing in correlation analyses, a Benjamini–Hochberg false discovery rate (FDR) correction was applied across a pre-specified subset of representative biomarkers capturing distinct anatomical and functional domains. Both unadjusted and FDR-adjusted *p*-values are reported. Because both eyes from each participant were included, inter-eye dependence was explicitly assessed. Intraclass correlation coefficients (ICCs) were calculated for all OCT, OCTA, and visual field parameters to quantify within-subject correlation. When ICCs demonstrated moderate to high inter-eye dependence (ICC ≥ 0.40), group comparisons and inferential analyses were conducted using linear mixed-effects models with participant identifier included as a random intercept. This modeling strategy appropriately accounts for within-subject clustering, provides valid estimation of standard errors, and reduces bias in parameter inference while preserving statistical power. All statistical tests were two-sided, and a *p*-value < 0.05 was considered statistically significant.

An a priori power analysis by using G*Power software (version 3.1.9.6) for an independent samples *t*-test with an anticipated effect size of 0.5 (Cohen’s d) and a significance level of α = 0.05 determined that a sample size of 51 eyes per group was required to achieve 80% statistical power for a two-tailed comparison.

## 3. Results

Overall, 120 eyes of 60 Caucasians patients (29 men, 31 women; mean age 34.40 ± 9.60 years) were included in the study analysis. Subjects were divided according to vitamin D level in group 1 (vitamin D levels < 30 ng/mL) and group 2 (vitamin D level > 30 ng/mL). There were no statistically significant differences between groups in terms of age (33.25 ± 9.20 vs. 35.55 ± 10.01 years, *p* = 0.192, Student’s *t*-test) and gender [14 males (46.7%) and 16 females (53.3%) vs. 15 males (50.0%) and 15 females (50.0%), *p* = 0.796, Chi-squared test]. Group 1 exhibited significant lower serum vitamin D levels compared with group 2 (18.29 ± 5.62 ng/mL vs. 37.89 ± 4.82 ng/mL, *p* < 0.01). The clinical characteristics of the groups are displayed in Table 1.

Spherical equivalent, intraocular pressure, and contrast sensitivity did not differ significantly between the two groups. Specifically, mean spherical equivalent was +0.42 ± 1.40 D in Group 1 and +0.12 ± 1.25 D in Group 2 (*p* = 0.056), while intraocular pressure averaged 15.25 ± 3.25 mmHg and 15.75 ± 3.42 mmHg, respectively (*p* = 0.297). Contrast sensitivity was comparable between groups, with values of 1.78 ± 0.16 logMAR in Group 1 and 1.83 ± 0.12 logMAR in Group 2 (*p* = 0.202), indicating no meaningful functional difference.

The OCT and OCTA measurements of the participants are summarized in Table 2.

Group 1 showed a significantly thinner central macular thickness (CMT) compared with Group 2 (267.66 ± 13.31 µm vs. 274.69 ± 14.96 µm; *p* = 0.035). Likewise, mean central macular point thickness was lower in Group 1 (280.12 ± 17.25 µm) than in Group 2 (289.56 ± 18.05 µm; *p* = 0.038). In contrast, paracentral macular thickness and all sectoral macular measurements (superotemporal, inferotemporal, superonasal, inferonasal) did not differ significantly between groups (*p* > 0.05 for all).

Analysis of the ganglion cell complex (GCC) revealed a significant reduction only in the inner inferior nasal sector in Group 1 compared with Group 2 (70.7 ± 13.14 µm vs. 76.45 ± 12.12 µm; *p* = 0.030). No significant differences were observed in the remaining inner or outer GCC sectors, nor in global superior or inferior GCC thicknesses (*p* > 0.05).

Regarding retinal microvasculature, the deep capillary plexus (DCP) demonstrated consistently lower perfusion density in Group 1 across all regions, including whole (40.19 ± 2.45% vs. 43.14 ± 3.12%; *p* < 0.001), inner (39.80 ± 2.10% vs. 42.25 ± 2.19%; *p* < 0.001), and outer regions (50.25 ± 2.05% vs. 54.11 ± 2.41%; *p* < 0.001). In contrast, superficial capillary plexus (SCP) perfusion showed more limited differences: while the inner SCP (41.14 ± 3.85% vs. 42.97 ± 4.05%; *p* = 0.027) and outer SCP (54.90 ± 4.08% vs. 57.90 ± 4.12%; *p* = 0.009) were reduced in Group 1, the whole SCP did not differ significantly between groups (*p* = 0.065).

Finally, although the foveal avascular zone (FAZ) area was numerically larger in Group 1 (0.35 ± 0.14 mm^2^) compared with Group 2 (0.30 ± 0.10 mm^2^), this difference did not reach statistical significance after accounting for inter-eye correlation (*p* = 0.168).

VF parameters of the two groups are summarized in Table 3.

Visual field–derived parameters were largely comparable between the two groups. Foveal threshold, hill of vision at 10° (HOV@10°), visual quality index (VQI), mean deviation (MD), mean deviation of the hill (MDh), pattern standard deviation (PSD), and most sectoral sensitivity measures did not differ significantly between groups (all *p* > 0.05).

A significant difference was observed for retinal sensitivity decline, which was more pronounced in Group 1 (−2.89 ± 1.29 dB) compared with Group 2 (−2.16 ± 1.04 dB; *p* = 0.003). In addition, mean central sensitivity was lower in Group 1 (25.09 ± 2.12 dB) than in Group 2 (25.70 ± 2.45 dB; *p* = 0.010).

By contrast, paracentral sensitivity and quadrant-specific sensitivities (superotemporal, superonasal, inferotemporal, and inferonasal) showed no statistically significant differences between groups (*p* > 0.05 for all comparisons), indicating preserved global visual field performance despite localized differences.

To limit inflation of Type I error due to multiple, partially collinear comparisons, a Benjamini–Hochberg false discovery rate (FDR) correction was applied to a pre-specified subset of nine representative biomarkers. These variables were selected a priori to capture distinct anatomical and functional domains, including retinal microvascular perfusion (Deep Capillary Plexus, Superficial Capillary Plexus), global macular structure (Central Macular Thickness), and visual function (retinal sensitivity decline, contrast sensitivity).

Redundant or highly collinear sub-sectoral parameters—such as individual ganglion cell complex quadrants or perfusion sublayers strongly correlated with whole-slab metrics—were intentionally excluded from the multiplicity adjustment to preserve statistical power and maintain interpretability.

Following FDR correction, all primary associations of interest remained statistically significant, particularly those involving deep capillary plexus perfusion and retinal sensitivity decline, confirming that the observed findings were robust and not driven by chance due to multiple testing. Associations that were borderline at the unadjusted level (e.g., whole superficial plexus perfusion and contrast sensitivity) did not survive correction and were therefore interpreted conservatively (Table 4).

Furthermore, serum vitamin D levels and structural/functional parameters in the participants were tested for a possible correlation (Table 5).

Serum vitamin D levels showed weak-to-moderate positive correlations with selected structural and microvascular retinal parameters. Specifically, vitamin D concentrations were positively correlated with central macular thickness (r = 0.27, 95% CI 0.033–0.474; *p* = 0.035) and mean central macular point thickness (r = 0.26, 95% CI 0.043–0.483; *p* = 0.038).

A stronger association was observed with deep capillary plexus perfusion, as vitamin D levels correlated positively with whole DCP vessel density (r = 0.45, 95% CI 0.310–0.657; *p* < 0.001). In contrast, the correlation with superficial capillary plexus perfusion did not reach statistical significance (r = 0.24, 95% CI −0.036–0.486; *p* = 0.065).

No significant association was detected between serum vitamin D levels and FAZ area (r = −0.18, 95% CI −0.391–0.038; *p* = 0.168).

Regarding functional parameters, vitamin D levels were positively correlated with retinal sensitivity decline at 10° (r = 0.37, 95% CI 0.155–0.564; *p* = 0.003), indicating that lower vitamin D levels were associated with greater sensitivity loss. However, no correlations were observed between vitamin D levels and global visual field indices beyond this parameter.

Overall, these correlations indicate modest associations with structural thickness and deep retinal perfusion, while relationships with superficial vascular metrics and FAZ area were weaker and did not achieve statistical significance.

## 4. Discussion

The present study investigated the relationship between serum vitamin D levels and retinal structural/functional parameters in healthy individuals with full visual acuity. The results revealed that vitamin D insufficiency is associated with statistically detectable but subclinical and focal structural variations in retinal microarchitecture and microvascular perfusion assessed through OCT and OCTA, despite the absence of clinically overt ocular pathology. Overall, visual function presented a more subtle scenario, with trends toward reduced contrast sensitivity and a steeper hill of vision, although visual field testing and Pelli–Robson contrast sensitivity remained within normal ranges in vitamin-D-deficient individuals.

Vitamin D deficiency is a widespread public health problem, affecting more than half of the population in several European and Asian countries [1,13,14]. Vitamin D participates in a broad spectrum of biological processes relevant to ocular health, including modulation of immune responses, protection against oxidative stress, neuroprotection, and regulation of angiogenic pathways [15,16,17]. The retina is particularly sensitive to vitamin D status: vitamin D receptors and vitamin-D-dependent calcium-binding proteins are expressed in retinal ganglion cells, the inner nuclear layer, photoreceptors, endothelial cells, and pericytes [18,19], providing a mechanistic substrate for structural and microvascular changes linked to hypovitaminosis D.

In this context, our findings show that individuals with serum vitamin D levels below 30 ng/mL exhibit small retinal alterations despite full best-corrected visual acuity and no clinically apparent ocular disease. CMT and adjacent macular measurements were reduced in vitamin-D-insufficient subjects, in agreement with previous OCT studies reporting thinner macular thickness in both young adults and elderly individuals with low vitamin D levels [20,21]. These anatomical changes align with epidemiological evidence suggesting an association between inadequate vitamin D levels and increased risk of AMD. Several large population studies have demonstrated that lower vitamin D levels are associated with early AMD [22,23,24], a reduced risk of AMD progression in postmenopausal women with higher serum concentrations [25], and an overall increased risk of late AMD based on meta-analytic data [8].

The neuroretinal findings observed, including focal thinning of the GCC, despite limited to inferior nasal sector, may reflect early metabolic stress in regions with dense neuronal populations and high energy demand. Indeed, studies on glaucoma have demonstrated that inferior retinal sectors may exhibit earlier structural changes, potentially due to regional differences in metabolic demand, vascular architecture, or cellular susceptibility to oxidative and inflammatory stress [26,27]. However, given the relatively large standard deviations compared to the observed mean differences in our cohort, the signal-to-noise ratio in these measurements is admittedly low, and these findings should be interpreted with appropriate caution. Nonetheless, experimental studies indicated that vitamin D reduced ganglion cell loss, attenuated oxidative damage, and modulated inflammatory cascades in models of retinal degeneration or optic neuritis [14,15]. Large epidemiologic analyses in healthy cohorts have also shown tight correlations between inner retinal thickness and visual performance, supporting the concept that structural thinning may precede measurable functional impairment [28,29].

Microvascular changes have emerged as a second major component of the retinal phenotype associated with low vitamin D levels. Perfusion density in the deep capillary plexus (DCP) was lower in vitamin D–insufficient eyes across whole, inner, and outer regions, whereas the superficial capillary plexus (SCP) showed regional reductions (inner and outer), with the whole SCP difference not reaching significance. Although the absolute magnitude of these differences was modest, our use of 3 high-resolution 3 × 3 mm scans may have allowed the detection of focal parafoveal perfusion deficits that may represent early subtle microvascular dysregulation. These findings are biologically plausible given that calcitriol modulates endothelial nitric oxide synthase (eNOS), reduces inflammatory cytokine production (IL-6, IL-8, TNF-α), inhibits pathological neovascularization, and protects endothelial cells against oxidative injury [16,17,30]. Existing OCTA literature remains heterogeneous: some studies describe increased DCP density as a potential compensatory response [31,32], whereas others report decreased perfusion in vitamin-D-deficient subjects [21,33]. Variability across scan sizes, imaging devices, and demographics likely contributes to these discrepancies.

FAZ area did not differ significantly between groups after accounting for inter-eye correlation in the mixed-effects analyses, despite a numerically larger mean value in vitamin-D-insufficient eyes. This lack of statistical separation, together with the wide overlap between group distributions, suggests that FAZ enlargement should not be interpreted as a meaningful anatomical alteration in this healthy cohort. FAZ measurements are known to be particularly sensitive to segmentation variability, motion artifacts, and physiological factors, which may further limit their reliability in detecting subtle systemic associations. In contrast, perfusion density metrics—especially those derived from the deep capillary plexus—showed more consistent and robust differences between groups, supporting their greater sensitivity as indicators of early microvascular changes associated with vitamin D status. Functional assessment revealed a selective and largely subclinical pattern. Global visual field indices—including foveal threshold, hill of vision at 10° (HOV@10°), mean deviation (MD), hemifield mean deviation (MDh), pattern standard deviation (PSD), and visual quality index (VQI)—were comparable between vitamin-D-insufficient and sufficient groups, indicating preserved overall visual field performance. However, eyes with lower vitamin D levels exhibited a greater retinal sensitivity decline at 10° and a modest reduction in mean central sensitivity, whereas paracentral and quadrant-specific sensitivities remained unaffected. This dissociation suggests a localized vulnerability of central macular function rather than a diffuse visual field impairment.

Importantly, contrast sensitivity measured with the Pelli–Robson chart did not differ between groups, confirming preserved global visual performance and supporting the interpretation that functional involvement remains subtle in this healthy cohort. Previous studies in early diabetic retinopathy and neuro-ophthalmic conditions have shown that contrast sensitivity and localized macular sensitivity may decline before overt defects appear on standard automated perimetry or structural imaging [11,34,35]. In this context, vitamin D may influence ganglion cell metabolism, mitochondrial function, and inflammatory signaling, providing a plausible biological substrate for early, focal functional changes without widespread impairment.

Taken together, these findings support a cautious structure–function framework in which subtle structural and microvascular alterations—particularly involving the central macula and deep capillary plexus—may precede or occur independently of clinically meaningful functional loss. In otherwise healthy individuals, the functional consequences of hypovitaminosis D therefore appear limited, localized, and remain below conventional detection thresholds, underscoring the subclinical nature of the observed associations.

Collectively, these structural, vascular, and emerging functional findings raise important clinical considerations. Vitamin D deficiency is highly prevalent worldwide; thus, early retinal changes such as those observed here may contribute to reduced neurovascular resilience over time. Interventional evidence supports this hypothesis: vitamin D supplementation has been shown to improve outcomes in diabetic macular edema and enhance responses to anti-VEGF therapy [36]. Similarly, the simultaneous delivery of resveratrol and metformin, has showed to prevent loss of endogenous antioxidants, and suppress the growth of abnormal vessels in the retina with macular degeneration [37]. If these nutritional agents influence retinal homeostasis, their optimization may represent a simple, modifiable strategy to preserve long-term retinal integrity. Furthermore, OCT- and OCTA-derived biomarkers such as CMT, GCC thickness, and SCP/DCP perfusion may offer a non-invasive means of identifying individuals with suboptimal nutritional status.

This study suffers from some limitations that deserve mentioning. Its cross-sectional nature prevents causal inference, and although adequately powered for structural comparisons, the sample size may not detect subtle functional deficits. Indeed, a critical limitation is the absence of functional impairment corresponding to the structural and microvascular findings. Despite comprehensive visual field testing and contrast sensitivity assessment, no clinically meaningful functional deficits were detected, with the exception of one isolated parameter (retinal sensitivity decline at 10°) among many comparisons. This structure-function discordance limits interpretation of the biological and clinical significance of our structural observations. Furthermore, restricting enrollment to young, healthy Caucasian adults with normal BMI strengthens internal validity but reduces generalizability. In addition, participants were not stratified into deficient, insufficient, and sufficient subgroups, which may have revealed different dose–response relationships. OCTA metrics are also susceptible to physiological variability, segmentation issues, and device-specific artifacts.

Nonetheless, by integrating serum vitamin D quantification with multimodal retinal imaging—including high-resolution OCT and OCTA—alongside visual field and contrast sensitivity testing, this work provides a comprehensive overview of how vitamin D insufficiency may manifest in the retina before clinical disease becomes apparent. The identification of early microstructural and microvascular alterations underscores the potential importance of vitamin D as a modifiable systemic factor in ocular health.

## 5. Conclusions

Low serum vitamin D levels were associated with subtle but measurable alterations in macular structure, ganglion cell integrity, and retinal microvascular perfusion in otherwise healthy young adults. These early changes—detectable through high-resolution OCT and OCTA—occurred in the absence of overt clinical disease or significant functional impairment, suggesting that hypovitaminosis D may contribute to a more vulnerable retinal microenvironment even before visual symptoms arise. The findings remain exploratory and association-based, supporting future longitudinal validation rather than diagnostic application.

The patterns of GCC thinning and reduced parafoveal capillary perfusion observed in vitamin-D-insufficient individuals mirror findings from previous experimental and epidemiologic studies reporting neuroprotective, anti-inflammatory, and vasculotropic functions of vitamin D. Although functional testing remained largely within normal ranges, the presence of early anatomical and vascular differences reinforces the possibility that vitamin D status may influence retinal resilience over time.

These observations, while preliminary, highlight the potential value of considering vitamin D status as a modifiable systemic factor relevant to retinal health. They also suggest that multimodal retinal imaging may serve as a sensitive tool for detecting subclinical changes associated with systemic nutritional imbalance. Longitudinal and interventional studies are needed to determine whether optimizing vitamin D levels can stabilize or reverse these early alterations and to clarify the extent to which vitamin D contributes to long-term neurovascular homeostasis in the retina.

## Figures and Tables

**Table 1:** Clinical characteristics of the study groups. Participants were classified according to serum vitamin D levels into Group 1 (<30 ng/mL; 60 eyes) and Group 2 (≥30 ng/mL; 60 eyes). Inter-eye correlation for each parameter was quantified using the intraclass correlation coefficient (ICC). Group differences were evaluated using linear mixed-effects models (equivalent to subject-clustered *t*-tests for two-group comparisons), with participant identifier included as a random intercept. Serum vitamin D level was entered as a fixed-effect covariate in all models.

	Group 1 (n = 60)	Group 2 (n = 60)	ICC	*p*-Value
Spherical equivalent ± SD (Diopters)	+0.42 ± 1.40	+0.12 ± 1.25	0.65	0.056
Intraocular pressure ± SD (mmHg)	15.25 ± 3.25	15.75 ± 3.42	0.60	0.297
Contrast sensitivity ± SD (logMAR)	1.78 ± 0.16	1.83 ± 0.12	0.74	0.202

**Table 2:** Comparison of OCT and OCTA findings of the two groups. Initial inter-eye correlation was assessed using intraclass correlation coefficients (ICCs). Group differences were subsequently evaluated using Student’s *t*-tests implemented within linear mixed-effects models, incorporating subject-level random intercepts to adjust for inter-eye dependence.

	Group 1, Vitamin D Levels Under 30 ng/mL (n = 60 Eyes)	Group 2, Vitamin D Levels over 30 ng/mL (n = 60 Eyes)	ICC	*p*-Value
CMT (μm)	267.66 ± 13.31	274.69 ± 14.96	0.64	**0.035**
Whole RNFL (μm)	102.30 ± 5.92	101.88 ± 7.98	0.61	0.973
Mean central points (μm)	280.12 ± 17.25	289.56 ± 18.05	0.71	**0.038**
Mean paracentral points (μm)	322.98 ± 19.21	324.12 ± 21.24	0.65	0.783
Mean Superotemporal (μm)	317.23 ± 14.25	315.53 ± 15.21	0.57	0.625
Mean Inferotemporal (μm)	310.87 ± 13.25	310.71 ± 14.11	0.49	0.739
Mean Superonasal (μm)	315.14 ± 14.20	315.15 ± 12.21	0.50	0.292
Mean Inferonasal (μm)	309.38 ± 12.00	311.02 ± 11.87	0.58	0.109
FAZ Area (mm^2^)	0.35 ± 0.14	0.30 ± 0.10	0.76	0.168
	SCP			
Whole (%)	42.25 ± 2.98	43.75 ± 3.14	0.64	0.065
Inner (%)	41.14 ± 3.85	42.97 ± 4.05	0.71	**0.027**
Outer (%)	54.90 ± 4.08	57.90 ± 4.12	0.77	**0.009**
	DCP			
Whole (%)	40.19 ± 2.45	43.14 ± 3.12	0.66	**<0.001**
Inner (%)	39.80 ± 2.10	42.25 ± 2.19	0.60	**<0.001**
Outer (%)	50.25 ± 2.05	54.11 ± 2.41	0.68	**<0.001**
	GCC 0.5/1.5/3 mm			
Inner Nasal inferior (μm)	70.7 ± 13.14	76.45 ± 12.12	0.51	**0.030**
Inner Temporal inferior (μm)	74.12 ±12.12	78.55 ± 11.03	0.66	0.367
Inner Nasal superior (μm)	82.54 ± 16.51	79.13 ± 14.32	0.64	0.964
Inner Temporal superior (μm)	78.72 ± 14.42	76.74 ± 12.42	0.59	0.680
Outer Nasal inferior (μm)	123.50 ± 9.91	124.07 ± 5.56	0.62	0.278
Outer Temporal inferior (μm)	117.62 ± 10.81	116.63 ± 6.15	0.67	0.798
Outer Nasal superior (μm)	126.86 ± 7.78	125.04 ± 6.70	0.61	0.674
Outer Temporal superior (μm)	119.32 ± 7.43	117.55 ± 6.26	0.78	0.994
Superior (μm)	111.01 ± 7.89	108.81 ± 6.70	0.62	0.502
Inferior (μm)	107.12 ± 9.23	108.75 ± 5.06	0.61	0.600

CMT: central macular thickness; DCP: deep capillary plexus; SCP: superficial capillary plexus; FAZ: foveal avascular zone; RNFL: retinal nerve fiber layer; GCC: ganglion cell complex. Statistically significant results are shown in bold.

**Table 3:** Visual field (VF) findings of the two groups. Intraclass correlation coefficients (ICCs) were initially computed to assess the degree of inter-eye correlation. Group comparisons were subsequently conducted using Student’s *t*-tests within a linear mixed-effects modeling framework, incorporating a random intercept for each participant to adjust for within-subject dependence.

	Group 1, Vitamin D Levels Under 30 ng/mL (n = 60 Eyes)	Group 2, Vitamin D Levels over 30 ng/mL (n = 60 Eyes)	ICC	*p*-Value
Fovea threshold (dB)	27.21 ± 3.11	27.01 ± 3.29	0.49	0.647
HOV@10° (dB)	23.25 ± 1.74	23.15 ± 1.89	0.42	0.649
Decline (dB)	−2.89 ± 1.29	−2.16 ± 1.04	0.72	**0.003**
VQI (%)	98.79 ± 1.11	98.71 ± 1.01	0.61	0.301
MDh (dB)	−1.83 ± 1.73	−1.89 ± 1.37	0.58	0.739
PSD (dB)	1.70 ± 0.27	1.72 ± 0.44	0.69	0.900
MD (dB)	−0.28 ± 0.15	−0.24 ± 0.14	0.67	0.357
Mean Central (dB)	25.09 ± 2.12	25.70 ± 2.45	0.59	**0.010**
Mean Paracentral (dB)	23.85 ± 2.08	24.15 ± 1.89	0.63	0.275
Mean ST (dB)	23.78 ± 2.42	24.12 ± 2.24	0.55	0.659
Mean SN (dB)	23.26 ± 2.34	23.49 ± 2.27	0.70	0.758
Mean IT (dB)	23.49 ± 2.25	23.73 ± 2.14	0.62	0.272
Mean IN (dB)	23.63 ± 2.22	23.91 ± 2.32	0.64	0.742

HOV@10°: hill of vision at 10 degrees; VQI: vision quality index; MD: mean deviation; MDh: hemifield mean deviation; ST: superotemporal; SN: superonasal; IT: inferotemporal; IN: inferonasal. Statistically significant results are shown in bold.

**Table 4:** Benjamini–Hochberg false discovery rate (FDR) correction (q = 0.05) was applied to nine pre-specified representative biomarkers spanning structural, vascular, and functional domains. These included Deep Capillary Plexus whole/inner/outer perfusion (%), Superficial Capillary Plexus inner/outer perfusion (%), Central Macular Thickness (µm), retinal sensitivity decline at 10° (dB), and contrast sensitivity (log units). Parameters with unadjusted *p*-values below their corresponding FDR-adjusted thresholds were considered statistically significant (in bold).

Variable	Unadjusted *p*-Value	Critical Value (FDR 0.05)	Significance
DCP Whole	**<0.001**	**0.006**	**Significant**
DCP Inner	**<0.001**	**0.011**	**Significant**
DCP Outer	**<0.001**	**0.017**	**Significant**
Sensitivity Decline	**0.003**	**0.022**	**Significant**
SCP Outer	**0.009**	**0.028**	**Significant**
SCP Inner	**0.027**	**0.033**	**Significant**
CMT	**0.035**	**0.039**	**Significant**
SPC Whole	0.065	0.044	Not Significant
Contrast Sensitivity	0.090	0.050	Not Significant

**Table 5:** Correlations between serum vitamin D levels and selected structural and functional retinal parameters. Pearson’s correlation coefficients (r) with corresponding 95% confidence intervals (CIs) are reported. Correlation analyses were performed using subject-clustered models to account for inter-eye dependence.

		CMT	Mean CMT Point	Retinal Sensitivity Decline	DCP Whole	SCP Whole	FAZ
Vitamin D serum levels	Correlation coefficient (r)	0.27	0.26	0.37	0.45	0.24	−0.18
	95% Confidence Interval	0.033–0.474	0.043–0.483	0.155–0.564	0.310–0.657	−0.036–0.486	−0.391–0.038
	*p*-value	**0.035**	**0.038**	**0.003**	**<0.001**	0.065	0.168

r: Pearson’s correlation test; CMT: central macular thickness; DCP: deep capillary plexus; SCP: superficial capillary plexus; FAZ = foveal avascular zone. Statistically significant results are shown in bold.

## Data Availability

The original contributions presented in this study are included in the article. Further inquiries can be directed to the corresponding author.
